# Mucosal-Associated Lymphoid Tissue Lymphoma in Southeast Asia: A 15-Year Retrospective Multicenter Study

**DOI:** 10.3390/hematolrep17060063

**Published:** 2025-11-25

**Authors:** Kannadit Prayongratana, Tanapun Thamgrang, Chonlada Laoruangroj, Lalita Norasetthada, Thanawat Rattanathammethee, Udomsak Bunworasate, Kitsada Wudhikarn, Jakrawadee Julamanee, Panarat Noiperm, Suporn Chuncharunee, Pimjai Niparuck, Archrob Khuhapinant, Noppadol Siritanaratkul, Piyapong Kanya, Kanchana Chansung, Chittima Sirijerachai, Dusit Jit-Uaekul, Juthatip Chaloemwong, Nonglak Kanitsap, Peerapon Wong, Nisa Makruasi, Somchai Wongkhantee, Tawatchai Suwanban, Tanin Intragumtornchai

**Affiliations:** 1 Division of Hematology, Department of Medicine, Phramongkutklao Hospital and College of Medicine, Bangkok 10400, Thailand; dear_tanapun@hotmail.com (T.T.);; 2 Division of Hematology, Department of Medicine, Faculty of Medicine, Chiang Mai University, Chiang Mai 52000, Thailand; 3 Division of Hematology, Department of Medicine, Faculty of Medicine, Chulalongkorn University, Bangkok 10330, Thailandtanin4563@gmail.com (T.I.); 4 Division of Hematology, Department of Medicine, Faculty of Medicine, Prince of Songkla University, Songkhla 90110, Thailand; 5 Division of Hematology, Department of Medicine, Faculty of Medicine, Ramathibodi Hospital, Mahidol University, Bangkok 10400, Thailand; 6 Division of Hematology, Department of Medicine, Faculty of Medicine, Siriraj Hospital, Mahidol University, Bangkok 10700, Thailand; 7 Division of Hematology, Department of Medicine, Chiang Rai Prachanukroh, Chiang Rai 57000, Thailand; 8 Division of Hematology, Department of Medicine, Khon Kaen University, Khon Kaen 40002, Thailand; 9 Division of Hematology, Department of Medicine, Faculty of Medicine, Vajira Hospital, Bangkok 10300, Thailand; 10 Division of Hematology, Department of Medicine, Nakornping Hospital, Chiang Mai 50180, Thailand; 11 Faculty of Medicine, Thammasat University, Pathumthani 12121, Thailand; 12 Division of Hematology, Department of Medicine, Naresuan University, Phitsanulok 65000, Thailand; 13 Division of Hematology, Department of Medicine, Srinakharinwirot University, Nakhon Nayok 26120, Thailand; 14 Division of Hematology, Department of Medicine, Khon Kaen Regional Hospital, Khon Kaen 40000, Thailand; 15 Division of Hematology, Department of Medicine, Rajavithi Hospital, Bangkok 10400, Thailand

**Keywords:** mucosal-associated lymphoid tissue lymphoma, MALT lymphoma, extranodal marginal-zone lymphoma, marginal-zone lymphoma

## Abstract

**Objective:** To describe the epidemiology, survival rate, and prognostic factors of mucosal-associated lymphoid tissue (MALT) lymphoma. **Patients and Methods:** This investigation utilized the Thai Lymphoma Study Group (TLSG) registry to gather data on patients diagnosed with MALT lymphoma. The analysis included demographic details, therapeutic interventions, and survival statistics. **Results:** The TLSG registry prospectively included 8404 patients with lymphoma. Among them, marginal-zone lymphoma (MZL) was the second most common subtype, with 670 histologically confirmed cases, accounting for 8.0% of the total cohort. An analysis of the MZL subtypes showed that MALT lymphoma was the most common, accounting for 77.8% of the diagnoses. This was followed by nodal MZL at 17.5% and splenic MZL at 7.7%. The distribution of primary disease sites indicated that the ocular adnexa (49.2%), stomach (12.9%), and sinonasal region (12.5%) were the three most common locations. Three variables were found to be statistically significant predictors of survival in the multivariate analysis: ECOG performance status > 2, age exceeding 65 years, and involvement of more than two extranodal organs. These identified prognostic factors were further assessed for their effect on overall survival (OS) and progression-free survival (PFS). A risk classification was established: the low-risk group comprised patients with zero identified risk factors, whereas the high-risk group included patients who had any of the specified risk factors. A comparison of five-year survival rates showed significantly more favorable outcomes for low-risk patients who had a PFS of 83.3% (vs. 66.1%, *p* = 0.028) and an OS of 97.8% (vs. 76.7%, *p* < 0.001) compared to the high-risk group. **Conclusions:** In this cohort, where MZL was the second most common lymphoma and MALT lymphoma was the predominant subtype, our analysis revealed that patients with no risk factors experienced statistically significant improvements in both PFS and OS.

## 1. Introduction

Marginal-zone lymphoma (MZL) is the second most common indolent B-cell lymphoid malignancy after follicular lymphoma, accounting for approximately 6% of all lymphoma cases [1]. According to the 5th edition of the World Health Organization (WHO) Classification of Tumours of Haematopoietic and Lymphoid Tissues, MZL is classified into four distinct subtypes: extranodal marginal-zone lymphoma (EMZL), also known as mucosa-associated lymphoid tissue (MALT) lymphoma; nodal marginal-zone lymphoma (NMZL); splenic marginal-zone lymphoma (SMZL); and primary cutaneous marginal-zone lymphoma (PCMZL) [2,3].

MALT lymphomas typically arise from chronic antigenic stimulation of specific organs. In Western countries, the stomach is the most prevalent site of involvement, primarily due to *Helicobacter pylori* infection. However, in developing countries, the pattern of organ involvement may differ, likely due to variations in antigenic exposures, with both infectious and autoimmune conditions being more prevalent [4,5,6,7].

Non-gastric MALT lymphomas are also linked to chronic infections or autoimmune diseases. For example, MALT lymphoma of the ocular adnexa is associated with *Chlamydophila psittaci*, small intestinal MALT lymphoma with *Campylobacter jejuni*, cutaneous MALT lymphoma with *Borrelia burgdorferi*, and bronchial MALT lymphoma with *Achromobacter xylosoxidans.* Autoimmune conditions such as Sjögren’s syndrome and Hashimoto’s thyroiditis are associated with MALT lymphomas of the salivary/lacrimal glands and thyroid gland, respectively [8].

Genetic aberrations are also found in MALT lymphomas from various sites. For instance: t(11;18)(q21;q21)/*API2::MALT1* is most frequent in MALT lymphoma of the lung (45%), followed by the stomach (23%) and intestine (19%); t(1;14)(p22;q32)/*BCL10::IGH* is found in MALT lymphoma of the lung (8%), intestine (7%), and stomach (2%); t(14;18)(q32;q21)/*IGH::MALT1* occurs in the ocular adnexa (16%), skin (7%), and salivary glands (6%); t(3;14)(p14.1;q32)/*FOXP1::IGH* has been reported in MALT lymphoma of the thyroid (50%), ocular adnexa (20%), and skin (10%) [9,10,11].

According to data from the Thai Lymphoma Study Group (TLSG), which analyzed 4056 lymphoma patients nationwide, MZL was the second most common subtype after diffuse large B-cell lymphoma (DLBCL) and represented the most prevalent indolent lymphoma, accounting for 6.2% of all cases [12]. Among patients with MZL, extranodal MZL or MALT lymphoma was the most frequent subtype, followed by nodal MZL (NMZL) and splenic MZL (SMZL). In the present study, we expanded the cohort to include twice as many patients as in the previous analysis, with the aim of further characterizing the clinical features of MZL and developing a prognostic index specific to MALT lymphoma.

## 2. Materials and Methods

### 2.1. Study Design and Patient Population

This multicenter, retrospective study included patients registered in the TLSG database from 15 academic centers across Thailand between 2007 and 2022. All newly diagnosed NHL patients aged ≥ 15 years were enrolled in the registry. The TLSG database captures all lymphoma subtypes nationwide and collects comprehensive clinical, laboratory, and treatment-related data. For this study, we extracted data specifically for patients diagnosed with EMZL.

### 2.2. Data Collection and Variables

The following variables were evaluated: age, sex, primary site and number of extranodal organs involved, Eastern Cooperative Oncology Group (ECOG) performance status, Ann Arbor stage, hepatitis B virus (HBV) infection status, and serum lactate dehydrogenase (LDH) level. Treatment modalities, including rituximab-containing regimens, were also recorded. Data entry and pathological classification of lymphoma subtypes were performed locally at each participating center according to standard institutional practice. The pathological diagnosis of EMZL at each participating institution was based on the prevailing World Health Organization (WHO) classification criteria at the time of diagnosis.

### 2.3. Study Endpoints and Follow-Up

The study endpoints were progression-free survival (PFS) and overall survival (OS), defined according to the revised response criteria for malignant lymphoma [13]. OS was measured from the date of diagnosis to death from any cause. PFS was defined as the time from diagnosis to disease progression or death, whichever occurred first. Multivariable analyses were conducted to identify independent prognostic factors and to develop predictive models for PFS and OS based on baseline clinical variables.

### 2.4. Ethical Considerations

The study was approved by the Institutional Review Board (IRB) of each institution. All procedures were conducted in accordance with the principles of the Declaration of Helsinki and the International Conference on Harmonisation (ICH) guidelines for Good Clinical Practice. Due to the retrospective and de-identified nature of the data analysis, a waiver of patient informed consent was granted by the IRB.

### 2.5. Statistical Analysis

The minimum required sample size for developing the prognostic model was estimated using the “pmsampsize” approach for survival data [14]. Based on the study by Juan Pablo Alderuccio et al., the discriminative ability of the MALT-IPI was represented by Harrell’s C-statistic, reported to be approximately 0.7 for both PFS and OS [15]. We planned to include five candidate predictors. An expected event rate of 20%, derived from a 5-year OS of 80% in a previous study [12], and a conservative Cox–Snell R^2^ of 0.1 were assumed, with a shrinkage factor of 0.9 to minimize model overfitting. Assuming a mean follow-up duration of 3 years, the minimum required sample size was 425 patients, corresponding to 850 person-years of follow-up and 170 outcome events, given an overall event rate of 0.2 and an events-per-predictor parameter (EPP) of 34.

Continuous variables are presented as mean ± standard deviation (SD) or median with range or interquartile range (IQR), as appropriate. Categorical variables are expressed as frequencies and percentages. Univariate Cox proportional hazards regression was performed to identify factors associated with PFS and OS. Variables with *p*-value < 0.20 in the univariable analysis were included in the multivariable model to avoid excluding potentially important prognostic factors [16]. The proportional hazards assumption was verified using Schoenfeld residuals. Kaplan–Meier survival curves were generated, and differences between risk groups were assessed using the log-rank test. Because we included only patients with complete data, no imputation of missing data was necessary, as all variables used in the multivariate Cox regression analyses were fully complete.

All statistical analyses were performed using Stata software, version 17.0 (StataCorp LLC, College Station, TX, USA). All *p*-values are two-sided, with *p*-value < 0.05 considered statistically significant.

## 3. Results

From January 2007 to December 2022, a cohort of 8404 lymphoma patients was recruited from 15 medical institutions across the nation for the TLSG registry of the 8404 lymphoma patients, 670 (8.0%) received a histological diagnosis of MZL. This positions MZL as the second most common lymphoma subtype, preceded by DLBCL. Regarding MZL subtypes, MALT lymphoma was the predominant entity, identified in 497 patients (74.2%). In descending order of frequency, this was followed by NMZL (117 patients, 17.5%), SMZL (52 patients, 7.7%), and PCMZL (4 patients, 0.5%). After excluding individuals with incomplete follow-up, 457 patients from the MALT lymphoma population were included in the long-term survival analyses.

**Figure 1:**
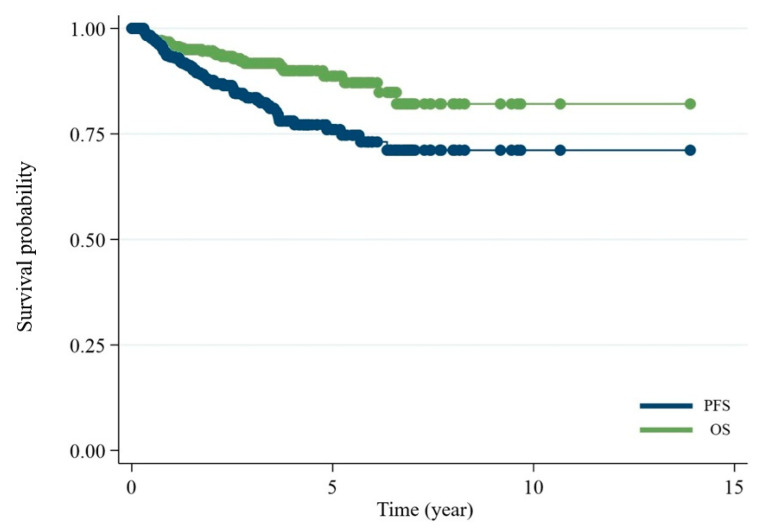
Kaplan–Meier curves showing overall survival (OS) and progression-free survival (PFS) for all patients in the cohort. The 5-year PFS was 76.4% and 5-year OS was 89.3%.

The three most common primary sites of involvement were the ocular adnexa (*n* = 225; 49.2%), stomach (*n* = 59; 12.9%), and sinonasal region (*n* = 57; 12.5%). Baseline patient characteristics are summarized in Table 1. The median age at diagnosis was 59 years (range: 20–95), with nearly one-third of patients (*n* = 162; 35.4%) aged 65 years or older. There was a slight male predominance (56.9%). An ECOG performance status of 3–4 was observed in 15 patients (3.5%). Involvement of two or more extranodal sites was noted in 78 patients (17%), while advanced-stage disease (Ann Arbor stage III–IV) was present in 162 patients (35.4%). Elevated lactate dehydrogenase (LDH) levels were found in 112 patients (24.5%). One hundred and three patients (22.5%) had B-symptoms. For treatment modality, chemotherapy (CMT) alone was the treatment for 126 patients (27.6%), CMT combined with rituximab (R) in 61 (13.3%), CMT + radiation (RT) in 53 (11.6%), R + CMT + RT in 10 (2.2%), R monotherapy in 2 (0.4%), and antibiotic eradication in 30 (6.6%). Locoregional therapy with RT was administered to 111 patients (24.3%), while surgical resection alone was performed in 16 patients (3.5%). With a median follow-up of 25 months (IQR 11–42 months), the 5-year OS and PFS rates were 88.2% and 76.3%, respectively (Figure 1).

Univariate analysis identified a higher number of extranodal sites, poor ECOG performance status, and advanced stage (III–IV) disease as factors associated with inferior PFS. In multivariate analysis, having ≥2 extranodal sites remained the only statistically significant factor associated with inferior PFS (adjusted hazard ratio [aHR] 2.13; 95% confidence interval [CI]: 1.25–3.62; *p* = 0.005).

In the univariate analysis, age ≥ 65 years, an increased number of extranodal sites, poor ECOG performance status (ECOG 3–4), advanced disease stage (III–IV), and the presence of B symptoms were all associated with inferior OS (Table 2). In the multivariate analysis, only age ≥ 65 years (aHR 2.48, 95% CI: 1.18–5.21; *p* = 0.017) and ECOG performance status 2–4 (aHR 3.50, 95% CI: 1.57–7.79; *p* = 0.002) remained independent predictors of inferior OS (Table 3).

Univariate Cox regression analysis was conducted to evaluate the impact of gender, age, primary site of MALT lymphoma, number of extranodal sites involved, ECOG performance status, hepatitis B virus serostatus, LDH level, and Ann Arbor stage. Age ≥ 65 years, ECOG ≥ 2, involvement of ≥2 extranodal organs, presence of B symptoms, Ann Arbor stage III–IV, and elevated LDH levels were identified as potential factors associated with progression-free survival (PFS) or overall survival (OS). Variables with a *p*-value < 0.2 were included in the multivariate analysis (Table 2). Three variables—age ≥ 65 years, number of extranodal sites ≥ 2, and ECOG ≥ 2—were retained in the final model, which was used to construct the prognostic index (Table 3).

**Figure 2:**
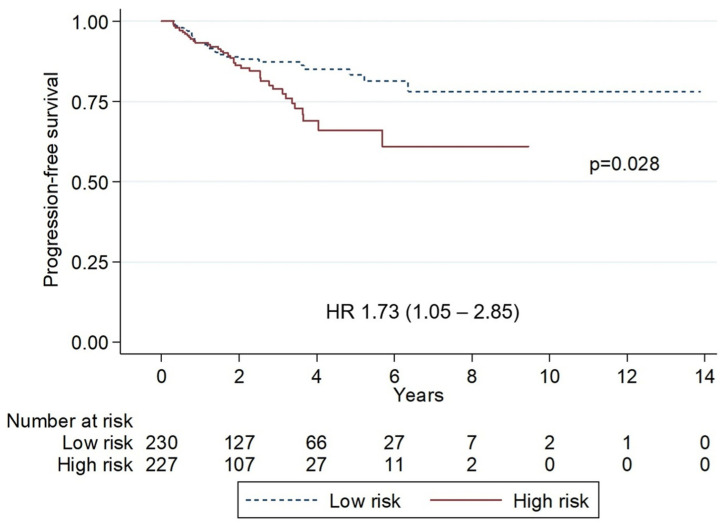
Kaplan–Meier curves of progression-free survival stratified by risk groups. The low-risk group showed superior survival, 5-year progression-free survival 83.3% vs. 58.7% (*p* = 0.028). HR, hazard ratio.

**Figure 3:**
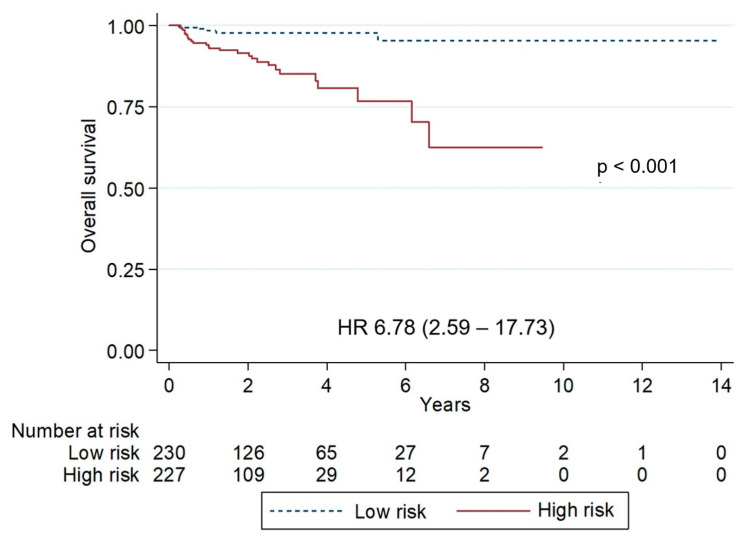
Kaplan–Meier curves of overall survival stratified by risk groups. The low-risk group showed superior survival, 5-year progression-free survival 97.8% vs. 72.7% (*p* < 0.001).

Patients without any adverse prognostic factors for OS identified in the multivariate analysis were classified as low-risk, whereas those with one or more risk factors were categorized as high-risk. The five-year PFS was significantly lower in high-risk patients compared with low-risk patients (66.1% vs. 83.3%; *p* = 0.028; HR 1.73, 95% CI: 1.05–2.85). Similarly, five-year OS was markedly lower in high-risk patients (76.7% vs. 97.8%; *p* < 0.001; HR 6.78, 95% CI: 2.59–17.73) (Figure 2 and Figure 3).

## 4. Discussion

Our study, conducted between January 2007 and December 2022 and encompassing 8404 lymphoma patients, identified MZL as the second most common subtype after DLBCL), accounting for 8% of all cases. This prevalence was higher than that reported in a previous Thai study on non-Hodgkin lymphoma, which found MZL to comprise 6.2% of cases, following DLBCL at 58.1% [12]. Similar findings have been reported in other Asian countries, including China and Korea, where MZL was the most common indolent B-cell lymphoma subtype [17,18]. Among MZL subtypes in our cohort, MALT lymphoma constituted the majority (74.2%).

This study represents the first large-scale analysis to elucidate the epidemiology and prognosis of MALT lymphoma in Thailand. Typically, the most common primary organ involvement of MALT lymphoma is stomach (30–40%), followed by ocular adnexa [6,18]. However, in our cohort, ocular adnexa was the most common primary organ involvement (49.2%), followed by stomach (12.9%) and sinonasal area (12.5%). In the previous report of the TLSG study, ocular adnexa was also the most common site of involvement [12]. The distribution of MALT lymphoma appears to vary among different countries due to differing chronic antigen stimulation, which is the core pathogenesis of MALT lymphoma Gastric lymphoma, which has been linked to chronic *Helicobacter pylori* infection, has also been found to decrease in recent years due to *H. pylori* eradication [4].

A pathogenetic model of antigen-driven lymphoproliferation, similar to that proposed for *H. pylori*-related gastric MALT lymphomas, has been suggested for ocular adnexa MALT lymphoma (OAML). This notion is supported by the association between OAML and *Chlamydophila psittaci* infection [19]. The DNA of *C. psittaci*, an obligate intracellular pathogen, has been detected in 80% of OAML patients in one study [20]. The prevalence of *C. psittaci* infection in OAML patients varies among different reported studies, and geographical variability in this association has been indicated, with prevalence rates of positive *C. psittaci* infection in OAML ranging from 0% to 100% [19]. However, these variations could also be explained by methodological pitfalls as well as by the effect of some confounding factors such as the use of wide-spectrum antibiotics and the involvement of other antimicrobial agents. In a report in Thailand, 121 OAML patients exhibited a good response to treatment, especially radiotherapy, with excellent long-term outcomes. The five-year PFS and OS rates were as high as 66.1% and 94.0%, respectively [21]. The relationship between *C. psittaci* infection and OAML in Thailand remains unexplored in published literature. A study examining the association between *C. psittaci* infection, autoimmune disease, and OAML is currently in progress.

MALT lymphoma affected adults across a wide age range (median 59 years, range 20–95), consistent with previous reports [2,7,18]. One-third of patients (35.4%) were aged ≥ 65 years, and males were slightly predominant (56.9%). In contrast to U.S. data showing female predominance at certain sites, males remained predominant in Thailand, even among ocular adnexal cases (60.4%) [6,22].

Treatment modalities for MALT lymphoma varied according to the site and stage of disease. In our cohort, chemotherapy alone was the most common approach (27.5%), while the use of rituximab, either as monotherapy or in combination with other treatments, was relatively low—likely reflecting limited access to rituximab among Thai patients. As this was a registry-based study, treatment heterogeneity and data completeness were key limitations. Nevertheless, previous retrospective studies have shown that the choice of initial treatment modality—whether chemotherapy, surgery (alone or combined), or radiotherapy—did not significantly influence survival outcomes in MALT lymphoma [23,24,25]. In the future, prospective study should be conducted to state benefit of each treatment modality.

A prognostic tool for MALT lymphoma, the MALT Lymphoma International Prognostic Index (MALT-IPI) [26], was developed and validated using data from 401 patients prospectively enrolled in the IELSG-19 randomized study [27], in which gastric MALT lymphoma was the most common site of involvement (42.6%). The MALT-IPI stratified patients into three prognostic groups—low, intermediate, and high risk—based on the presence of 0, 1, or 2–3 of the following factors: age ≥ 70 years, stage III–IV disease, and elevated LDH levels. The corresponding five-year PFS rates were 56.8%, 48.0%, and 22.7% for the low-, intermediate-, and high-risk groups, respectively.

In contrast, our model categorized patients into two groups—low and high risk—based on age ≥ 65 years, involvement of ≥2 extranodal sites, and ECOG performance status ≥ 2. In our cohort, the five-year PFS for high-risk patients was 66.1%, which exceeded that of low-risk patients in the MALT-IPI model. Furthermore, analysis of the Surveillance, Epidemiology, and End Results (SEER) database identified age ≥ 60 years as an independent predictor of shorter lymphoma-specific survival in patients with localized EMZL, suggesting that a lower age cutoff may be more appropriate for risk stratification [28].

Interestingly, although LDH is a key component in many prognostic indices for non-Hodgkin lymphoma [29], it did not significantly influence PFS or OS in our model. This discrepancy may reflect differences in disease characteristics or subtype distributions of MALT lymphoma across populations. Consequently, the applicability of the MALT-IPI and other existing prognostic models to Thai patients warrants further validation to determine their predictive accuracy in this population.

The main limitation of this study lies in its retrospective design, which relied on data from the TLSG registry. As a result, some patients could not be included in the analysis due to incomplete data. Furthermore, a centralized histopathological review was not performed to confirm the diagnosis for each case, introducing the potential for misclassification of lymphoma subtypes.

In addition to baseline prognostic indices used to predict initial outcomes, emerging evidence suggests that disease progression within two years of initiating therapy (POD24) is associated with inferior survival in MALT lymphoma [30,31]. Therefore, future studies should investigate the association between POD24 and clinical outcomes among Thai patients with MALT lymphoma.

## 5. Conclusions

Our study demonstrated the clinical characteristics, prognostic factors, and treatment outcomes of Thai MALT lymphoma patients, showing that ocular adnexa MALT lymphoma is the most common subtype. Furthermore, we developed a prognostic index for Thai MALT lymphoma using simple variables to identify high-risk patients. Individuals with one or more of the following risk factors—age ≥ 65 years, ECOG ≥ 2, or involvement of ≥2 extranodal sites—are expected to have lower PFS and OS. As this study lack of data association between *C. psittaci* and ocular adnexa lymphoma, future research should investigate the association between these, as well as validate our prognostic model in larger cohorts and explore novel therapeutic strategies tailored to high-risk patients. In addition, the impact of POD24 on survival outcomes should be further evaluated.

## Figures and Tables

**Table 1:** Patient characteristics.

Characteristic		Number	%
Age	Median, year (range)	60 (17–95)	
	<65 years	295	64.6%
	≥65 years	162	35.4%
Gender	Female	197	43.1%
	Male	260	56.9%
Type of EMZL	Ocular adnexa	225	49.2%
	Stomach	59	12.9%
	Sinonasal	57	12.5%
	Salivary gland	19	4.2%
	Thyroid	16	3.5%
	Lung/Pleura	24	5.3%
	Breast	8	1.8%
	Small intestine	16	3.5%
	Large intestine	9	2.0%
	Skin/Subcutaneous	4	0.9%
	Others	5	1.1%
	Missing	15	3.3%
Number of extranodal sites	1	379	82.9%
	2	60	13.1%
	3	17	3.7%
	4	1	0.2%
ECOG	0	237	51.9%
	1	169	37.0%
	2	36	7.9%
	3	11	2.4%
	4	4	0.9%
B-symptoms	Presence	103	22.5%
	Absent	354	77.5%
HBV status (HBsAg or Anti-HBc Ab)	Positive	104	22.8%
	Negative	353	77.2%
Ann Arbor staging	I–II	295	64.6%
	III IV	162	35.4%
LDH	Normal	345	75.5%
	High	112	24.5%

**Table 2:** Univariate analysis of adverse factors for PFS and OS.

Variables	PFS			OS		
	HR	95%CI	*p*-Value	HR	95%CI	*p*-Value
Age						
≥65 years	0.93	0.54–1.59	0.792	3.27	1.60–6.68	0.001
Gender						
Male	1.02	0.63–1.66	0.935	1.53	0.74–3.17	0.255
Type of EMZL						
Ocular adnexa	1.04	0.64–1.69	0.882	0.54	0.26–1.12	0.096
Number of extranodal sites						
2	1.84	1.00–3.37	0.048	1.55	0.63–3.82	0.337
3–4	4.15	1.94–8.86	<0.001	3.07	0.91–10.30	0.070
ECOG						
1	1.53	0.90–2.60	0.117	1.45	0.62–3.44	0.393
2	1.73	0.71–4.17	0.225	2.67	0.85–8.40	0.093
3–4	5.46	1.89–15.75	0.002	22.08	8.32–58.58	<0.001
HBsAg or Anti-HBc Ab						
Positive	1.32	0.75–2.34	0.335	0.76	0.29–1.99	0.576
LDH						
High	1.33	0.76–2.31	0.319	1.95	0.94–4.07	0.074
Advanced stage						
Yes	1.77	1.09–2.88	0.022	1.73	0.86–3.48	0.121
B-symptoms						
Yes	1.59	0.94–2.69	0.085	2.10	1.03–4.30	0.043

**Table 3:** Adverse prognostic factors for PFS or OS according to multivariate analysis.

Variables	PFS			OS		
	HR	95%CI	*p*-Value	HR	95%CI	*p*-Value
Age ≥ 65 years	0.86	0.5–1.49	0.591	2.48	1.18–5.21	0.017 *
Number of extranodal sites 2–4	2.13	1.25–3.62	0.005 *	1.39	0.62–3.11	0.418
ECOG 2–4	1.65	0.81–3.38	0.169	3.5	1.57–7.79	0.002 *

* Denotes statistical significance (*p* < 0.05).

## Data Availability

The datasets generated and analyzed during the current study are not publicly available due to participant privacy concerns but are available from the corresponding author, Dr. Kannadit Prayongratana, upon reasonable request and subject to an institutional data use agreement.

## Abbreviations

The following abbreviations are used in this manuscript:

MALT	mucosal-associated lymphoid tissue
TLSG	Thai Lymphoma Study Group
MZL	Marginal-zone lymphoma
OS	Overall survival
PFS	Progression-free survival
EMZL	Extranodal marginal-zone lymphoma
NMZL	Nodal marginal-zone lymphoma
SMZL	Splenic marginal-zone lymphoma
PCMZL	Primary cutaneous marginal-zone lymphoma
DLBCL	Diffuse large B-cell lymphoma
ECOG	Eastern Cooperative Oncology Group
LDH	Lactate dehydrogenase
CMT	Chemotherapy
RT	Radiation
R	Rituximab
aHR	Adjusted hazard ratio
HR	Hazard ratio
CI	Confidence interval
HBsAg	Hepatitis B surface antigen
Anti-HBc Ab	anti-hepatitis B core antibody
OAML	Ocular adnexa MALT lymphoma
MALT-IPI	MALT Lymphoma International Prognostic Index
SEER	Surveillance, Epidemiology, and End Results
